# Pancreatic safety of DPP-4 inhibitors in type 2 diabetes millitus: A protocol for systematic review and network meta-analysis

**DOI:** 10.1097/MD.0000000000029154

**Published:** 2022-05-06

**Authors:** Fan Yang, Youzi Dong, Baohua Li, Bobiao Ning, Quanlin Zhao

**Affiliations:** a The First Clinical Medical College, Shandong University of Traditional Chinese Medicine, Jinan, Shandong, China; b Division of Comprehensive Internal Medicine, The Affiliated Hospital of Shandong University of Traditional Chinese Medicine, Jinan, Shandong, China.

**Keywords:** dipeptidyl-peptidase IV inhibitors, network meta-analysis, pancreatic safety, systematic review, type 2 diabetes mellitus

## Abstract

**Background:**

Dipeptidyl-peptidase IV inhibitor (DPP-4i) is a common hypoglycemic medication in treating type 2 diabetes millitus. It has become widely utilized in clinical practice due to its ability to effectively manage blood glucose while posing a low risk of hypoglycemia and weight gain. However, there is no consensus on DPP-4i's pancreatic safety due to a paucity of clinical evidence. The safe event appears to be easily overlooked. This review aims to evaluate the pancreatic safety of DPP-4i in patients with type 2 diabetes mellitus using the standard pairwise and network meta-analysis methods.

**Methods:**

MEDLINE, Embase, PubMed, Web of Science, and the Cochrane Central Register of Controlled Trials will be used to search for published literature on the pancreatic safety of DPP-4 inhibitors in type 2 diabetes millitus, and clinical trial registries will be used to look for unpublished trials. Two independent reviewers will screen literature for eligibility, extract available data, and assess the risk of bias. All divergences will be resolved after rechecking the source papers and further discussion among the reviewers with a complete consensus before inclusion. The risk of bias will be assessed by the Cochrane bias risk tool, and the quality of evidence will be interpreted by the GRADE Working Group approach. We will use STATA16.0 and WinBUGS1.4.3 for paired meta-analysis and Bayesian network meta-analysis.

**Results:**

This study will evaluate the pancreatic safety of DPP-4 inhibitors in type 2 diabetes millitus.

**Conclusion:**

This systematic review and network meta-analysis will evaluate the pancreatic safety of DPP-4i in patients with type 2 diabetes millitus. The findings of this study may supplement the evidence-based information on DPP-4i, improve existing understanding of this issue, and assist patients and clinicians in making better treatment decisions by raising their awareness of the problem.

**Protocol registration number:**

INPLASY202230014.

## 1 Introduction

Dipeptidyl-peptidase IV inhibitor (DPP-4i) is an incretin-based pharmacotherapy, a new antidiabetic drug that differs from conventional oral glucose-lowering medications. They decrease the inactivation of incretin hormones such as glucagon-likepeptide-1 (GLP-1) and gastric inhibitory polypeptide, raising the level of endogenously generated incretin hormones by inhibiting the dipeptidyl-peptidase IV, which degrades principal hormones into inactive products. Incretin hormones stimulate insulin secretion while inhibiting glucagon secretion in a glucose-dependent way, lowering blood glucose and reducing the risk of hypoglycemia and weight gain. Apart from the effects mentioned above, Dpp-4i has certain cardiovascular safety advantages that protect the heart and do not raise the risk of severe cardiovascular adverse events or mortality in type 2 diabetes millitus (T2DM) patients. As a result, this medication is frequently given to treat T2DM as a monotherapy or in conjunction with other hypoglycemic medicines.

In recent years, more attention has been devoted to the cardiovascular or renal outcomes of DPP-4i. However, other safety events, such as pancreatic events that are uncommon and controversial, are more likely to be overlooked. Clinically, related pancreatic events mainly refer to pancreatic exocrine diseases, such as pancreatitis and pancreatic cancer. According to the mechanism of action of DPP-4i, which increases GLP-1 in the body, some researchers hypothesized that GLP-1-based therapy could hasten the potential adverse effects of exocrine dysplasia and induce α-cell hyperplasia, which can negatively affect the pancreatic ducts and increase the risk of acute or chronic pancreatitis, as well as pancreatic cancer.^[1]^ An experiment that reveals increased pancreatic ductal turnover, ductal metaplasia, and isolated pancreatitis in rats treated exclusively with DPP-4i found a potential connection between DPP-4i treatment and pancreatic events.^[2]^ These findings could provide evidence for how incretin therapy contributes to pancreas-related disorders. Regrettably, other researchers do not share this viewpoint. The TECOS Study discovered no statistically significant difference between the Sitagliptin and placebo groups, while numerical differences exist.^[3]^ The population-based research of incretin-based treatment in Taiwan found no link between the DPP-4i and acute pancreatitis and the risk of pancreatic cancer in the short term.^[4]^ However, it is challenging to overcome restrictions imposed by technique and objective situations. The safety of DPP-4i medicines on the pancreas is still a worry for diabetic individuals. While DPP-4is have specific benefits for antidiabetic treatment, none of them could offset the slight risk of having unfavorable pancreatic consequences.

Numerous researchers have investigated this topic in vivo and vitro, and some secondary analysis has also been conducted, but no consensus has been reached based on the evidence gathered thus far. Moreover, most of the published meta-analyses were a pairwise comparison of 2 treatments, which might skew the results’ validity. As a result, our research aims to compile all randomized controlled trials that compare the pancreatic endpoints of DPP-4i therapy to other antidiabetic drugs or placebo in T2DM patients to assess the pancreatic safety of DPP-4i in a pairwise and network meta-analysis.

## 2 Methods

Preferred Reporting Items for Systematic Review and Meta-Analysis Protocols will be used to guide the protocol for this systematic review and network meta-analysis (NMA).^[5]^ A completed Preferred Reporting Items for Systematic Review and Meta-Analysis Protocols checklist was utilized to guarantee the quality of the protocol. In addition, the review will be published using the Preferred Reporting Items for Systematic review and Meta-Analysis extension statement for network meta-analysis.^[6]^

### 2.1 Study registration

Our protocol has been registered on the International Platform of Registered Systematic Review and Meta-Analysis Agreement (INPLASY), and the registration number is INPLASY202230014 (DOI number = 10.37766/inplasy2022.3.0014, URL = https://inplasy.com/inplasy-2022-3-0014/).

### 2.2 Inclusion criteria

#### 2.2.1 Types of participants

- Patients diagnosed with type 2 diabetes according to the criteria formulated by WHO.^[7]^
- Be over 18 years old.
- Patients have been treated with DPP-4is or other active antidiabetic medications.
- No restrictions on race, sex, nationality, disease severity,

#### 2.2.2 Types of interventions and comparators

We will take into account the research that evaluated the following therapies involving DPP-4is (mainly including Sitagliptin, Saxagliptin, Vildagliptin, Linagliptin, and Alogliptin) compared with placebo or other hypoglycemic drugs (like Biguanides, Sulfonylureas, Thiazolidinediones, α-glycosidase inhibitors, SGLT-2 inhibitor, and DLP-1 receptor agonist) in patients with T2DM.

#### 2.2.3 Outcomes

- The primary outcome of our study is pancreatic safety events, such as pancreatitis and pancreatic cancer.
- The secondary outcome of interest will include the change of pancreatic enzyme from baseline.

#### 2.2.4 Types of studies

Only randomized controlled trials (RCTs) will be enrolled in this review, which compared DPP-4i therapies with other active hypoglycemic medications or placebo in T2DM patients with a minimum intervention length of 4 weeks. Either blinding or open-label studies will be considered. Furthermore, the literature will only be available in English. Other types of studies, such as in vivo or in vitro studies, case reports, reviews, and non-RCTs, will be eliminated.

### 2.3 Databases and search strategy

Medline, Embase, PubMed, Web of Science, the Cochrane Central Register of Controlled Trials (CENTRAL) were searched from their inception dates to August 10, 2021. Clinical trial registries (such as www.ClinicalTrials.gov) were also searched for unpublished trials. Moreover, we will check the systematic reviews and meta-analyses to search for further relevant studies and contact the authors to obtain incomplete data. Two researchers will perform the search, and all discrepancies will be resolved by discussion with a third reviewer. To find qualifying studies in PubMed, we will use the following search strategy, and it will be tweaked for each database. Table 1 shows the search strategies and retrieval type which will be employed in this investigation.

**Table 1:** Search strategies for PubMed.

No	Retrieval type
#1	Diabetes Mellitus, Type 2 [Mesh Terms]
#2	Diabetes Mellitus, Type 2 [Title/Abstract] OR Diabetes Mellitus, Noninsulin-Dependent [Title/Abstract] OR Diabetes Mellitus, Ketosis-Resistant [Title/Abstract] OR Diabetes Mellitus, Ketosis Resistant [Title/Abstract] OR Ketosis-Resistant Diabetes Mellitus [Title/Abstract] OR Diabetes Mellitus, Non Insulin Dependent [Title/Abstract] OR Diabetes Mellitus, Non-Insulin-Dependent [Title/Abstract] OR Non-Insulin-Dependent Diabetes Mellitus [Title/Abstract] OR Diabetes Mellitus, Stable [Title/Abstract] OR Stable Diabetes Mellitus [Title/Abstract] OR Diabetes Mellitus, Type II [Title/Abstract] OR NIDDM [Title/Abstract] OR Diabetes Mellitus, Noninsulin Dependent [Title/Abstract] OR Diabetes Mellitus, Maturity-Onset [Title/Abstract] OR Diabetes Mellitus, Maturity Onset [Title/Abstract] OR Maturity-Onset Diabetes Mellitus [Title/Abstract] OR Maturity Onset Diabetes Mellitus [Title/Abstract] OR MODY [Title/Abstract] OR Diabetes Mellitus, Slow-Onset [Title/Abstract] OR Diabetes Mellitus, Slow Onset [Title/Abstract] OR Slow-Onset Diabetes Mellitus [Title/Abstract] OR Type 2 Diabetes Mellitus [Title/Abstract] OR Noninsulin-Dependent Diabetes Mellitus [Title/Abstract] OR Noninsulin Dependent Diabetes Mellitus [Title/Abstract] OR Maturity-Onset Diabetes [Title/Abstract] OR Diabetes, Maturity-Onset [Title/Abstract] OR Maturity Onset Diabetes [Title/Abstract] OR Type 2 Diabetes [Title/Abstract] OR Diabetes, Type 2 [Title/Abstract] OR Diabetes Mellitus, Adult-Onset [Title/Abstract] OR Adult-Onset Diabetes Mellitus [Title/Abstract] OR Diabetes Mellitus, Adult Onset [Title/Abstract]
#3	#1 OR #2
#4	Dipeptidyl-Peptidase IV Inhibitors [Mesh Terms]
#5	Dipeptidyl-Peptidase IV Inhibitors [Title/Abstract] OR Dipeptidyl Peptidase IV Inhibitors [Title/Abstract] OR DPP-4 Inhibitor [Title/Abstract] OR DPP 4 Inhibitor [Title/Abstract] OR Inhibitor, DPP-4 [Title/Abstract] OR DPP-IV Inhibitor [Title/Abstract] OR DPP IV Inhibitor [Title/Abstract] OR Inhibitor, DPP-IV [Title/Abstract] OR DPP-4 Inhibitors [Title/Abstract] OR DPP 4 Inhibitors [Title/Abstract] OR DPP-IV Inhibitors [Title/Abstract] OR DPP IV Inhibitors [Title/Abstract] OR Gliptin [Title/Abstract] OR Dipeptidyl Peptidase 4 Inhibitor [Title/Abstract] OR Dipeptidyl-Peptidase IV Inhibitor [Title/Abstract] OR Dipeptidyl Peptidase IV Inhibitor [Title/Abstract] OR Inhibitor, Dipeptidyl-Peptidase IV [Title/Abstract] OR Dipeptidyl-Peptidase 4 Inhibitor [Title/Abstract] OR Inhibitor, Dipeptidyl-Peptidase 4 [Title/Abstract] OR Dipeptidyl-Peptidase 4 Inhibitors [Title/Abstract] OR Dipeptidyl Peptidase 4 Inhibitors [Title/Abstract] OR Gliptins [Title/Abstract] OR DPP4 Inhibitor [Title/Abstract] OR Inhibitor, DPP4 [Title/Abstract] OR DPP4 Inhibitors [Title/Abstract] OR Sitagliptin [Title/Abstract] OR Saxagliptin [Title/Abstract] OR Vildagliptin [Title/Abstract] OR Linagliptin [Title/Abstract] OR Alogliptin [Title/Abstract]
#6	#4 OR #5
#7	Randomized Controlled Trial [Publication Type] OR RCT [Publication Type] OR Controlled Clinical Trial [Publication Type] OR randomized [Title/Abstract] OR controlled [Title/Abstract] OR placebo [Title/Abstract]
#8	pancrea∗ [Title/Abstract] OR safety [Title/Abstract]
#9	#3 AND #6 AND #7 AND #8

### 2.4 Study selection process

The retrieved results were imported into EndNoteX9 software, 2 reviewers will conduct literature screening independently. They will screen titles and abstracts of all the retrieved records to find potentially eligible studies and then examine the entire text to select studies that match the inclusion criteria. After rechecking the source papers, debate among the 2 reviewers, and adjudicating by the third, disagreements will be resolved with an entire consensus before inclusion. A Preferred Reporting Items for Systematic review and Meta-Analysis flow diagram will outline the research selection procedure and reasons for exclusions.^[8]^ If a study was published in duplicate, we would choose the version with the most detailed content and data.

### 2.5 Data extraction

We will ensure a standardized data extraction template in advance, and 2 investigators will independently extract the data from all eligible studies in duplicate. All data will be recorded in Microsoft Excel 2019 software. Any discrepancies will be resolved by consensus or arbitrated by the third.

#### 2.5.1 Data basic information

The following trial information will be extracted: title, study author, publication year, study design, sample size, types of intervention and control, background therapy, funding source, trial registration number, etc.

#### 2.5.2 Participants

Population characteristics containing mean age, gender proportions, racial, duration of disease, length of the trial, loss of follow-up, and baseline level of HbA1c will also be collected.

#### 2.5.3 Interventions

Intervention information to be collected mainly includes the treatment method, frequency, dose, treatment duration.

#### 2.5.4 Outcomes

What is more, the following outcome measures will be gathered: Relative Risk of pancreatic safety event (involving pancreatitis and pancreatic cancer) and post-intervention values or changes from baseline with corresponding standard deviations for the pancreatic enzyme. Additionally, if the supplied data is incomplete, we will contact the authors for more information.

### 2.6 Risk of bias assessment

Two assessors will assess the risk of bias and quality of all included studies, with the third reviewer participating in the discussion as needed. They will pilot numerous samples before the formal evaluation to get to an agreement on assessment standards. Cochrane risk of bias tool will be used to assess the risk of bias for trials concerning 7 points: the judgment of the random sequence generation, allocation concealment, blinding of participants and personnel, blinding of outcome assessment, incomplete outcome data, selective reporting, and other sources of bias, which are graded as low, high or unclear risk of bias.^[9]^ Furthermore, we will try to gather all the information related to this review to control publication bias, and if more than 10 trials are included, we will assess the reporting bias through a funnel plot.

### 2.7 Heterogeneity test

The *I*^2^ value will be used to measure the degree of heterogeneity. When *I*^2^ ≤ 50%, the heterogeneity is acceptable, we will choose the fixed-effects model. When *I*^2^ > 50%, that means there is significant heterogeneity in the study, and we will make sensitivity analysis, subgroup analysis and meta-regression to explore the source of heterogeneity. If heterogeneity still exists, we will choose the random-effects model.

#### 2.7.1 Sensitivity analysis

Sensitivity analysis will be conducted to check the robustness of the primary outcome. Several possible nodes, such as sample size, methodological weakness, and missing data, will be considered. If the heterogeneity is improved after excluding the studies with a high risk of bias, indicating that the study affects the robustness of the outcome. Oppositely, if the heterogeneity change is not significant, it indicates that the result is reliable.

#### 2.7.2 Subgroup analyses and meta-regression

We will conduct subgroup analysis and meta-regression to explore the source of heterogeneity if significant heterogeneity and sufficient data exist. Subgroup analyses will combine effect sizes for each subgroup from the perspectives of clinical or methodological heterogeneity, including age, duration of disease, length of the trial, loss of follow-up, the baseline level of HbA1c, and quality of the study. And in meta-regression, regression analysis will be used to explore the influence of some covariables on the merger effect in meta-analysis.

### 2.8 Data synthesis and analysis

A descriptive overview of available data will be compiled and reported, namely trial and population characteristics, interventions, results, and risk of bias evaluations. We will plot a network regarding the evidence structure of direct comparisons, in which the size of nodes will be proportional to the sample size of each intervention, and the thickness of the lines will be proportional to the cumulative number of RCTs for each pairwise comparison. We will also use the contribution plot to present the influence of each direct piece of evidence.^[10]^ Both traditional pairwise meta-analysis and the network meta-analyse will be conducted. All quantitative analyses will be carried out using Stata 16.0 (TX)^[11]^ and WinBUGS 1.4.3 (Imperial College and MRC, UK).^[12]^

#### 2.8.1 Pairwise meta-analyses

A random-effects model will be employed for traditional pairwise meta-analyses if the included studies are of heterogeneity. The *I*^2^ value will measure the degree of heterogeneity. Relative risk for dichotomous data will be calculated as effect measures with 95% confidence intervals, and continuous data will be presented as weighted mean difference with 95% confidence intervals. If different scales are involved in studies, we will use standardized mean differences to present continuous data to eliminate its effect on results. Besides, sensitivity analysis will be performed to validate the stability of the results or exclude studies with a high risk of bias.

#### 2.8.2 Network meta-analyses

NMA is an upgrade of classical meta-analysis, which can simultaneously combine direct and indirect analysis, estimate the efficacy of interventions through common comparators even if they have not been investigated head to head in randomized clinical trials, and thus analyze the effects of multiple interventions compared with each other.

Before the analysis, examine the assumptions of consistency, heterogeneity, and similarity of the included studies within and across connections in the entire network of interventions to determine whether direct and indirect evidence is reasonable.^[13]^ An approach of loop inconsistency is used to evaluate the presence of inconsistency in each closed loop in which an intervention effect measured using an indirect comparison is not equivalent to the intervention effect measured using direct comparison. The *I*^2^ statistic will quantify the global heterogeneity, and the Cochran Q test and its *P* value will also be used to evaluate the heterogeneity.^[14]^ The predictive interval graph and confidence interval will be plotted to present the influence of heterogeneity on each pairwise comparison. Furthermore, the similarity will be evaluated by comparing the critical clinical and methodological characteristics that can influence the effects of studies between 2 sets.

Suppose the above assumptions of consistency, heterogeneity, and similarity are reasonable. In that case, NMA will be performed in a random-effects model, by a Bayesian framework using Markov Chains Monte Carlo methods with WinBUGS 1.4.3 and Stata 16.0 software to synthesize all the available evidence.^[15]^ The results of all pairwise comparisons will be reported as appropriate effect values with 95% confidence intervals. The network geometry of interventions will be plotted to present their concise characteristics, while forest plots and contribution figures will be used to show the combined effect value.^[16]^ Besides, we will display values of the Surface Under the Cumulative Ranking curve for each intervention as well as rankings of effects.^[17]^ And convergence will be assessed by the Gelman Rubin statistic method and inspection of Monte Carlo errors.^[15]^

### 2.9 Quality assessment

The collected evidence will be interpreted by the GRADE Working Group approach for rating the quality of treatment effect estimates from NMA. According to GRADE, the assessment requires estimates from direct, indirect, and combined evidence from direct and indirect sources, as well as quality ratings for the direct and indirect comparisons,^[18]^ which are primarily used to assess the quality of each piece of evidence in 5 areas: limitation, imprecision, inconsistency, indirectness, and publication bias.

## 3 Discussion

Dpp-4i is widely used in clinical practice due to its sound hypoglycemic effect and safety, but there are concerns that its use may increase the risk of pancreatic events. Pancreatic events, such as pancreatitis and pancreatic cancer, are rare but real in DPP-4i safety events. Some authorities, such as the European Medicines Agency and Food and Drug Administration, issued a warning on pancreatitis for all available DPP-4is,^[19]^ but it has not yet been contraindicated. Some clinical studies suggest a significant association exists between pancreatic events and DPP-4i, and Dpp-4i using is related to increased risks of pancreatic cancer in patients with type 2 diabetes.^[20–22]^ The results of some systematic reviews and meta-analyses also support this viewpoint.^[23–25]^ Nevertheless, most of the present data have not suggested any negative trend,^[19,26–28]^ although they are insufficient to draw reliable conclusions. Generally, the correlation observed in trials, whether positive or negative, could be owing to low incidence, limited sample size, chance, or bias but merits further investigation. More importantly, in addition to the hazards of pancreatic events, the health status of the pancreas is strongly related to the development of T2DM, any possibility of increased risk on the pancreas should be taken seriously.

The current studies have not concluded the association between DPP-4i and pancreatic safety. Both the European Medicines Agency and Food and Drug Administration indicate that pancreatic events will continue to be considered a risk associated with DPP-4i until more data are available.^[29]^ Among numerous analytical methods, NMA can synthesize all available data for statistical processing through quantitative synthesis to further guide clinical decisions. In this way, our study will analyze and compare the pancreatic outcomes of different DPP4i treatments for T2DM, focusing on randomized controlled trials that could ensure the quality of studies to some extent, although it may limit the number of studies included. There are few NMAs in this direction presently, and most of them are typical pairwise meta-analyses. Therefore, it is meaningful to gather various direct and indirect evidence as comprehensively as possible to provide reliable recommendations for pancreatic safety and clinical medication of DPP4i.

## Author contributions

**Conceptualization:** Fan Yang, Quanlin Zhao.

**Data curation:** Bobiao Ning, Baohua Li.

**Formal analysis:** Fan Yang, Youzi Dong, Baohua Li.

**Methodology:** Youzi Dong, Bobiao Ning, Baohua Li.

**Software:** Youzi Dong, Bobiao Ning.

**Writing – original draft:** Fan Yang, Youzi Dong.

**Writing – review & editing:** Fan Yang, Quanlin Zhao.
